# Gender Perspective in Research on Interventions in Children with Experiences of Parental Gender-Based Violence: Application of GPIHR Criteria

**DOI:** 10.3390/ijerph182111047

**Published:** 2021-10-21

**Authors:** Benjamín Pereira-Román, Concepción López-Soler, María Vicenta Alcántara López

**Affiliations:** 1 Faculty of Psychology, University of Murcia, 30100 Murcia, Spain; clopezs@um.es (C.L.-S.); mavialcantara@um.es (M.V.A.L.); 2 Association for the Development of Mental Health in Children and Youth “I Want to Grow”, 30001 Murcia, Spain

**Keywords:** systematic review, gender perspective, intervention, children, gender-based violence

## Abstract

The aim of this study was to analyse the inclusion of a gender perspective (GP) in scientific production on interventions for a reduction in psychological distress in children who have experienced parental gender-based violence (CEXPGBV). To achieve this, a review of publications was carried out in the Web of Science, EBSCOhost, ProQuest and Cochrane Library databases. A total of 3418 records were found, and 44 items of research selected. For GP analysis, the questionnaire “Gender perspective in health research” (GPIHR) was applied and relationships with the terminology of violence were analysed, as well as the definition of term used, references to violence by men or received by women and the instruments used to assess these. Generally, the assessed studies do not contain a GP, since 70% of the GPIHR items were answered negatively. Likewise, 89% of research used general terms to refer to violence without referring to gender. These results show the importance of considering instruments such as GPIHR in both the planning and development of future research in order to avoid possible gender bias.

## 1. Introduction

### 1.1. Gender Perspective and Gender-Based Violence

Gender perspective (GP) is a theoretical approach with the main aim of analysing gender inequalities, with some of its central categories being socialisation and gender roles/practices, and power relations or domination systems—subordination between sexes [1]. As Rohlfs et al. [2] have stated, the application of GP in health research has become a requirement of good practice in different fields and professions. Both national and international organizations stress the need to include GP in health research to advance scientific quality, avoid bias, reduce inequality and advance equity in people’s health [3,4,5]. Likewise, as Vázquez-Recio [6] concludes after analysis of scientific production, the inclusion of GP has become an ethical position in research. In recent decades, despite an increase in promoting the advantages of including GP, its application remains scarce [7]. One mechanism for increasing methodological rigor and advancing science is criteria ensuring inclusion of GP in research. For this purpose, we found the validated questionnaire “Gender perspective in health research” (GPIHR) by Tomás et al. [8].

In the area of gender-based violence, including this perspective in the research context has become inevitable, as its own legal definition reflects the “manifestation of discrimination, the situation of inequality and the relations of power of men on women” [9]. This is reflected in the latest data from the macro-survey on violence against women 2019 [10], where one in every two women (57.3%) residing in Spain has suffered violence during their lives for the simple fact of being a woman. Likewise, 89.6% had children when the violent episodes took place, and 51.7% reported that these had witnessed or heard the violence against the mother and had suffered violence at the hands of the partner. According to estimates of this macro-survey, 1,678,959 children live in homes where the woman is currently suffering some form of violence from their partner (physical, sexual, control-based, emotional, financial or fear-based).

### 1.2. Children with Experiences of Parental Gender-Based Violence (CEXPGBV)

Regardless of the high number of children of women in gender-based violence contexts, there is no specific term to define this situation. In the scientific literature, we find this population defined as “exposed to” “intimate partner violence” or “domestic violence”. Nevertheless, childhood and adolescence development in contexts where this type of violence occurs, besides affecting children’s well-being, also affects their construction of gender dynamics, and helps to perpetuate the relationship dynamics of gender inequality. Therefore, we propose identifying this population as “Children with experiences of parental gender-based violence” (CEXPGBV), since we believe this identifies and expresses the relational complexity that this experience entails for these children. Likewise, this conceptualization considers relational aspects on the part of the children related to attachment theory, development theory, social learning theory, emotional security theory, and the Eco-Biological Theory of Development [11,12]. The parental/referential distinction is made since, apart from the fact the person attacked is always the mother, the aggressor may have different profiles. Most commonly it is the father; however, we also find aggressors toward the mother who do not fulfil a parental role, but do, however, act as a referential (mother’s partner).

### 1.3. Research on Gender-Based Violence

A key factor when analysing scientific production on gender-based violence and children is language. English is the predominant vehicular language due to quality standards for scientific publication [13]. Despite the adoption of the term “Gender-Based Violence” in the Declaration on the Elimination of Violence against Women of the United Nations in 1994 [14], and the current support of the European Council and European Commission to refer to this problem, in the research context, the terminology is diverse, nonspecific and varies over time. In analysis by López-Cepero et al. [15], it was found that, among different terms available to refer to gender-based violence in intimate partner relationships, “domestic violence” is most used in this research area. Likewise, Reed et al. [16], after analysing the terminology in scientific production, concluded with the need to recognise intimate partner violence as a gender problem in research, in order to progress toward reducing this highly prevalent form of abuse.

Including GP in research is one strategy which can enable this problem to become more visible. According to the compilation by Ferrer-Pérez and Bosch-Fiol [17], the recognition of these is:

*“(a) between men and women there have existed and still exist historical inequalities and discrimination that cause gender gaps, (b) certain power relations are established, which are generally favourable to men as a social group and discriminatory towards women and (c) these relations have been socially and historically constructed, they condition life and the roles played by women and men, cross the entire social fabric and are articulated with others (such as those derived from social class, ethnicity, age, sexual preference or religion).”* (p. 72)

Likewise, another study [18] suggests introducing GP in psychological research in situations of gender-based violence through critical analysis of the following points: (a) theoretical models based on gender-based violence, its causes and mechanisms; (b) sample selection method; (c) instruments selected for assessment; and (d) carrying out meta-analytical reviews to learn the real scope of differences between abused and non-abused women and between abusers and non-abusers.

### 1.4. Sex/Gender in Research

In spite of the difference between “sex” and “gender”, both concepts tend to be used interchangeably in research. In the review by Ritz et al. [19] this conceptual distinction is clearly stated:

*“Sex can be thought of as a biological attribute (such as those characteristics relating to genetics, physiology, anatomy, or reproduction) used to classify sexually reproducing animals (typically as males or females), while gender refers to the social processes that collectively influence the social roles, relationships, behaviors, power, or other traits that are culturally accorded to those classified as women/girls and men/boys.”* (p. 4)

At a behavioural level, gender is expressed through practices or roles where the individual demonstrates a series of socially expected characteristics, whether for men or women [20]. In this regard, certain behaviour associated with the feminine, such as caring being expected from women, and self-sufficiency from men. However, there can also be a “crossed” function (a man with feminine characteristics or a woman with masculine) and “androgynous” functions (both masculine and feminine characteristics) [21,22]. It must be noted that these practices are not static and vary with time and culture, though power dynamics are perpetuated.

Therefore, using the term “gender” as a synonym for “sex” can cause bias in results interpretation, as the sample’s socialization factors are not being considered. In CEXPGBV, this becomes a crucial element in research, given that they are immersed in a family environment with maximum expression of male dominance over women. Along these lines, different studies show that in gender-based violence dynamics, the aggressors usually fulfil the male gender role [23,24,25] and use parenting models which perpetuate gender stereotypes [26,27].

It should be mentioned that in the present work, binary terms are used (man–woman; masculine–feminine), though this dichotomization is artificial as these are social constructs, and do not reflect diversity of sexes and possible genders derived from interactions between biological and social factors [19].

### 1.5. Impact on CEXPGBV and Differences by Sex/Gender

There are several systematic reviews and meta-analyses highlighting the psychological distress caused to children who have witnessed gender-based violence towards their mother in the primary family nucleus [28,29,30]. Data from these studies are consistent with the latest report by the Government Delegation for Gender Violence (2015) “The invisible victims of gender violence” in the Spanish population [31], where we found that these children present statistically significant difficulties related to internalizing responses (concordant with anxiety processes, depression, somatic complaints), externalizing (breach of norms/limits, aggressive behaviour) and post-traumatic stress disorder (PTSD), with several comorbidities among these, defined as Complex Trauma or Developmental Trauma [32,33].

As for differences in the impact on this population according to the variable “sex”, at the start of the 21st century, externalizing symptoms were associated with boys, and internalizing with girls [34]. However, based on subsequent reviews, the data are inconclusive. On the one hand, we found meta-analyses where results express a greater association of externalizing symptoms in boys, but not in terms of internalizing symptoms in girls [28,35]. On the other hand, analyses by Kitzmann et al. [36] concluded that sex did not play a moderating role in internalizing or externalizing symptoms, and similarly Vu et al. [37] did not find that sex was associated with exposure to gender violence and adaptation problems in childhood. Finally, in the review by Fong et al. [38], they conclude with a trend linking externalizing/aggressive behaviour and the sex of the children with the sex of the aggressive parent. It must be noted that in all studies mentioned, sex and gender were used as synonyms despite being conceptually different study variables.

There is less scientific production in research that takes gender into account. In fact, we found no meta-analyses or systematic reviews where differences in the involvement of CEXPGBV with GP are analysed. We did find studies by Smagur et al. [39] where the effect of gender roles was assessed as a predictor of internalizing and externalizing behaviour problems in this population at 4 years of age. In the starting hypotheses, behavioural problems were expected according to gender roles; however, it was found that girls with gender roles typified as “feminine” showed a risk of externalizing, but not internalizing, behaviour problems. These results are explained due to the detrimental effects of assuming femininity as inferior. Likewise, in their analysis they presented possible bias in these results as mothers and fathers have difficulty in observing internalisation problems in this age group [40,41,42].

### 1.6. Intervention in CEXPGBV

Since the 1980s, several studies have been published where different types of child–adolescent psychological interventions are presented and used to reduce psychological distress in children who have witnessed violence towards their mothers [34]. In the last two decades, several systematic reviews and meta-analytic analyses on interventions for CEXPGBV have been published. Analyses of these are mainly directed toward methodology used in interventions (individual, family, group and joint). As for gender analysis, in the meta-analysis by Romano et al. [43] where five meta-analyses and seven systematic reviews were analysed, they state a lack of data (or inadequate data) which limits examination of the weight of basic demographic variables such as sex or variables that might be relevant (e.g., attitudes toward family violence) as key moderators in identifying the long-term effects of intervention.

We found a recent meta-analysis by Latzman et al. [44], which included randomised controlled trials (RCTs) considered of low or moderate risk of bias, finally totalling eight studies for analysis (*n* = 924). In this study, no mention was made of sex or gender in analyses regarding children, nor its possible moderating effect on results. It only referred to the children’s relationship with the aggressor and parent who had been attacked, stating that, in 7 out of 8 selected studies, the assaulted person was the mother; in the remaining study the assaulted parents were fathers and mothers. It is striking that in analysis of this situation, the only study whose sample of assaulted parents is mixed is what the author terms the most “inclusive”, adding no further reference to the matter. However, no reference is made to the percentage of assaulted mothers and fathers in the sample of this article, nor consideration that in 87.5% of selected studies the attacked parent is the mother (therefore, at the population level, this study rather than being “inclusive” is actually damaging to the sample), nor is there analysis explaining the reasons for difference between abused fathers and mothers, or whether there exist gender factors which explain this.

### 1.7. Study Aim

The aim of this study was to analyse the inclusion of a gender perspective in scientific production available until 2020 on interventions to reduce psychological distress in children who have suffered parental/referential gender-based violence.

The aims are: (1) to identify published experimental, quasi-experimental, non-experimental and single-case studies of intervention in the reduction of psychological distress in CEXPGBV; (2) quantify and describe variables related to gender and violence in research (terminology, definition, assessment); (3) assess inclusion of a gender perspective in published studies using the GPIHR instrument; and (4) identify possible relationships between variables described and GPIHR dimensions.

## 2. Materials and Methods

A systematic review was performed to achieve these aims; the writing of this research was carried out following recommendations from the Preferred Reporting Items for Systematic reviews and Meta-Analyses (PRISMA) statement [45] and considerations for professional practice by Sánchez-Meca and Botella [46].

### 2.1. Search Strategy

Given the socio-sanitary nature of the present study, the Web of Science, EBSCO host, ProQuest and Cochrane library meta-databases were used to search for research. A total of 46 databases were selected with a health and/or social nature, as well as general research. The Mendeley bibliographic manager was employed to manage the search information. The selection of keywords for each search element (see Appendix A Table A1) was through a conceptual bibliographic review in terms of terminology used in research on gender-based violence, prevalence of psychopathological involvement in CEXPGBV and interventions aimed at this population.

### 2.2. Inclusion and Exclusion Criteria

The search was restricted to studies published up to 2020, compiling all previous published material. Inclusion criteria were: (1) experimental studies (RCTs), quasi-experimental studies, non-experimental and single-case studies; (2) the attacked parent being a woman; (3) intervention aims to counteract the effects of violence on the mental health of the children, regardless of modality; (4) the study had to be written in English. Research was excluded where: (1) the intervention was directed at mothers/fathers without assessing the impact on children; (2) children were in child protection centres; (3) if the aim of intervention was different to counteracting the consequences of experiencing parental/referential gender-based violence.

### 2.3. Data Extraction

For each study, the term used to describe violence was compiled and categorised as “general” if not referring to gender (or associated factors) or as “specific” if doing so. Likewise, it was analysed whether the term of violence used was described, whether it refers to such violence by men or received by women and whether any instrument is used to assess it, either in frequency, intensity or duration. In addition, year of publication of study and decade were gathered to analyse temporal evolution.

### 2.4. Gender Perspective Assessment

GP analysis in studies was performed using the questionnaire “Gender perspective in health research” (GPIHR) by Tomás et al. [8], where items were adapted to childhood research (Appendix A Table A2). This comprises 10 items: 3 assess introduction, 1 aims, 3 methodology and 3 the research aim. The GPIHR has three factors revealing different levels of GP inclusion in research projects:

- Factor 1: Gender sensitivity. Referring to differences in health between men and women and the relationship between gender factors and the health issue addressed in the research project.
- Factor 2: Feminist research. Gathering all necessary conditions for research to have a gender perspective and a feminist purpose, i.e., investigating the causes of inequality in order to try and change them.
- Factor 3: Sex difference. Reflecting disaggregation of data by sex and age group, enabling identification of differences in health.

Completion of the GPIHR questionnaire was performed in a dichotomous “yes/no” manner for each question. It was considered “Yes” when the question could be answered in the affirmative with the information provided in the assessed research. It was considered as “No” when the evaluated research did not provide the necessary information to answer the question, when the information provided was ambiguous or directly led to a negative answer.

All GPIHR questions are focused on the inclusion of GP-related factors in the research. Therefore, the greater the number of “Yes” responses in the questionnaire, the greater gender perspective the research is considered to contain.

### 2.5. Statistical Analysis

Results analysis was carried out through the statistical program IBM SPSS by descriptive analysis, the Kolmogorov–Smirnov test was used to verify the assumption of normality of data in the GPIHR, the Kruskal–Wallis test for analysis of categorical variables with more than two categories, the Mann–Whitney U for categorical variables with two categories, and Spearman’s rank-order correlation coefficient to quantify the relationship between ordinal variables. When a statistically significant result was obtained with the Kruskal–Wallis test, two-to-two posterior comparisons were made with the Mann–Whitney U test. In this case, in order to control inflation of Type I error rate, the Bonferroni correction was applied on probability values to achieve a statistically significant result at a nominal significance level of 5%, and *p* value associated with the U test result had to be equal to or less than 0.017.

## 3. Results

### 3.1. Study Selection

**Figure 1:**
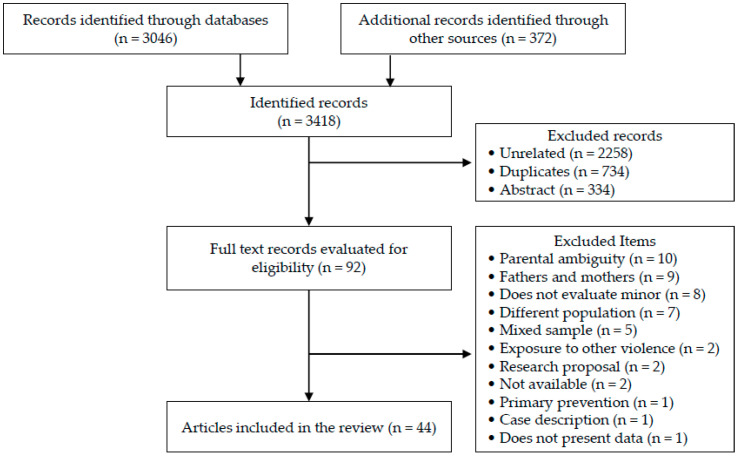
Flow diagram.

A total of 3418 records were obtained, 89% identified through database searches, 11% other sources (Figure 1). Forty-four records were finally included [47,48,49,50,51,52,53,54,55,56,57,58,59,60,61,62,63,64,65,66,67,68,69,70,71,72,73,74,75,76,77,78,79,80,81,82,83,84,85,86,87,88,89,90]. It should be noted, of 48 records excluded in the last screening phase, 39.6% related to the ambiguity of describing as “parent” the main caregiver of the children who had been assaulted (*n* = 10), since the groups of people attacked were a mixture of fathers and mothers (*n* = 9). In all studies where groups of the abused parent were mixed, the vast majority were women, with percentages ranging between 81% and 98% [91,92,93,94,95,96,97,98]. Only one study [99] does not provide specific data. However, it reports that in 65% of cases the physical aggressor was the father, 2.5% the mother and 32.5% both parents. Furthermore, it reports that in 87% of cases the mother reported the child’s state. Likewise, of all selected studies, in only one was the intervention aimed at the father and assessed consequences in the children.

### 3.2. Study Characteristics

The systematic review showed that, from 44 studies selected, 42% were RCTs (*n* = 19), 29% quasi-experimental (*n* = 13), 27% non-experimental (*n* = 11) and 2% single cases (*n* = 1). Table 1 shows frequency analysis. As for the terminology of violence used in the research, general terms were most used (89%). Intimate Partner Violence and Domestic Violence were those most used, in 39% and 32% of studies, respectively, the remainder (19%) being general terms with few repetitions (marital violence, domestic abuse, family violence, violent homes and traumatic violence). A total of 11% of studies referenced some factors related to gender (woman abuse, battered woman and wife abuse), of which two used the term gender- based violence.

### 3.3. GPIHR Questionnaire Results

In the Appendix A (Table A3), we found compliance of GPIHR items for each selected study. For frequencies of compliance with the GPIHR questionnaire in selected studies (Table 2), items were generally answered favourably 30% of the time. By factor, 18% of items related to “Gender sensitivity”, 8% to “Feminist research” and 68% to “Sex difference” were answered favourably. For analysis of the normal distribution of data in the GPIHR, the Kolmogorov–Smirnov test was employed, showing a significance of 0.005; therefore, distribution was not normal.

### 3.4. Relationship between Variables and GPIHR

Not having normal distribution meant the non-parametric Kruskal–Wallis test was used to analyse categorical variables of study type if the concept of violence used refers to gender, if the study expresses the gender component in the violence and if the violence and decade in which it was published are assessed. As shown in Table 3, significant differences were seen regarding whether the study assesses violence in the “Sex difference” dimension and in the decade of the study both in the general score of the GPIHR and in the “Feminist research” dimension.

For the variables in which significant values were found, the Mann–Whitney U test was employed, comparing whether there were differences 2 to 2. Of all the a posteriori comparisons made, statistically significant differences were found in the decade of study, both in total score of GPIHR and in the “Feminist research” dimension. As regards the total score, significant differences were found between the studies published up to 2000 and those between 2001 and 2010 (*p* = 0.023), with a higher average of scores in the first period. Likewise, significant differences were found between the decade from 2001 to 2010 and from 2011 to 2020 (*p* = 0.036), the mean score being higher in the most recent decade. Additionally, as for the “Feminist research” factor, significant differences were found when comparing the categories “Up to 2000” and “From 2001 to 2010” (*p* = 0.008), the average range being higher in the studies published up to 2000 than in those between 2001 and 2010. Finally, significant differences were also found in the scores in the “Sex difference” factor depending on whether the study assessed violence towards the mother (*p* = 0.035), finding higher mean scores in the studies where violence was assessed.

Spearman’s correlation coefficients were calculated (Table 4) to assess if there were correlations between ordinal variables. Significant positive correlations were seen between year of publication of study and that the study defines the term of violence used and assesses violence towards mothers that children have experienced. Likewise, a significant positive relationship was observed between the variables “definition of the term of violence used” and “expression in the investigation of the sex component in intimate partner violence”. Additionally, a significant negative correlation was found regarding assessing violence and the experimental model. The latter was coded with value 1 for RCT, 2 for quasi-experimental, 3 for non-experimental and 4 for a single case. In this way, the more current the study, the greater the tendency for the methodology used to be considered of greater statistical validity.

As for interactions of GPIHR scores with the rest of the selected variables, a significant positive correlation was only found for the “Sex difference” dimension with the variables “Year” and “Assessment of violence”. In other words, it was observed that the higher the scores in “Sex difference”, the higher the assessment of violence received by the mother.

Likewise, it has been found that assessing violence towards the mother is significantly related to more recent studies, with higher methodological quality and with the variable “Sex difference” in the GPIHR. These results point to a tendency in current studies to consider the severity and typology of experiences of aggression towards mothers, as well as the quality of internal validity in the experimental design, with variables such as age, sex and sex difference in results.

Finally, positive correlations were observed in all GPIHR dimensions with each other, except for “Sex difference” and “Feminist research”. This shows a trend where higher scores in any dimension make them more expected in other dimensions, except for “Sex difference” and “Feminist research”.

## 4. Discussion

The fact that the study generally takes gender in violence into account, that the term of violence used includes a gender factor, or that it assesses violence is not related to the GPIHR instrument. We only found a significant direct correlation with the variables “Year” and “Assessment of violence” with the GPIHR dimension “Sex difference”. However, this correlation between the two suggests it might be due to methodological factors inherent to the study rather than factors related to gender perspective.

Among elements which help to explain these results, we find the low GP scores of the studies (explaining why there is no normal distribution of results) and the number of studies where the assessed variables were taken into account. By dimension, we found that from items of the “Gender sensitivity” dimension, 18% of occasions were answered positively, in the “Feminist research” dimension only 8% and in “Sex difference”, 68%. Nevertheless, for study variables, we found that only 11% (*n* = 5) used a term of violence with a gender component, 41% (*n* = 18) presented the sex component in intimate partner violence (more assaulted women or men who were aggressors) and 64% (*n* = 28) assess violence between parents in some way.

### 4.1. Factor “Sex Difference” (Items 5, 6 and 8)

Bearing in mind the content of items of the factor with the highest completion rate (“Sex difference”), we find this refers to processes related to basic methodology factors. Thus, we found high compliance rates of 84% and 95% due to the stratification of the sample by sex and age, respectively. However, only 25% of studies show or mention analysis by sex in their study results. It should be noted that there are no significant differences between study methodology and performance of these analyses. These data agree with the findings of the meta-analysis by Romano et al. [53], where the lack of data (or inadequate data) do not allow us to analyse the weight of differences in the intervention in basic variables such as sex.

### 4.2. Factor “Gender Sensitivity” (Items 1, 2, 4 and 7)

Despite low compliance in “Gender sensitivity” (18%), analysing item by item, the highest compliance is in the introduction referring to magnitude of the problem in boys and girls (30%). Next, 23% of research on methodology highlighted the relationship between the problem (internalizing, externalizing responses, etc.) and some gender factors. Among the most assessed variables, we found attitudes and beliefs about violence toward children; however, knowledge about abuse dynamics, behaviour in the face of violence towards their mother and self-blame were also seen. It should be noted that the same variable can be considered a gender factor in some studies, but not in others. One example is that of Muela et al. [77], where it is specified that they study possible relationships between sex and externalizing responses from a gender perspective and as an intervention mediator. Nonetheless, in the rest of the studies that take type of response and sex into account, the response was not considered a gender factor as it was not examined from the child’s socialisation. Similarly, the parenting variable was included as a gender factor in analysis by Jouriles et al. [66], given that it is seen as a moderating factor in results from aspects related to care, gender and impact of violence. In other selected studies, despite assessing aspects regarding parenting, a gender factor was not taken into account as it was not considered from a socialisation viewpoint.

The “Gender sensitivity” factor item referring to the search for association between the health problem studied and some gender determinant through aims or hypotheses, was only met in 14% of studies (*n* = 6). We found that, based on the literature, Graham-Bermann et al. [56], hypothesised that the moderating variable “disclosure” of traumatic experiences in therapeutic groups would occur more in girls than in boys. Likewise, Muela et al. [77] hypothesised that boys would present greater clinical symptoms than girls, particularly externalizing. On the other hand, one hypothesis of Jouriles et al. [66] is that sex would moderate the relationship between frequency of contact with the mother’s aggressor and behavioural problems, predicting a greater relationship in girls than in boys. In the case of Hiltz-Hymes [60] and Pernebo et al. [79], they state among their aims the exploration of sex as a moderator in interventions, and Jaffe et al. [63] described their objective as exploring stereotypes and myths related to the male/female sex in the sample. Finally, the item regarding mention in the introduction of the study as to whether there was scientific literature with a gender perspective only appeared in 4% of research (*n* = 2). Specifically, Hiltz-Hymes [60] refers to research stating that psychopathology together with boys’ early exposure to violence can increase the likelihood that a person accepts and/or uses patriarchal ideologies to rationalise or justify their abusive or violent behaviour. On the other hand, Jouriles et al. [66] presents different studies explaining how socialization factors cause girls to be more affected than boys, deriving from continuous contact with their mother’s violent partner.

### 4.3. Factor “Feminist Research” (Items 3, 9 and 10)

The factor with the lowest compliance was “feminist research” (8%), which brings together items aimed at researching the causes of inequality with the aim of changing it. Only three studies consider gender category in the introduction as a determinant in the mental health of these children (item 3). Hiltz-Hymes [60] expresses how the ecological approach considers the influence of patriarchal structures, parental characteristics, sex, age and other factors as an explanation for children’s responses to parental gender violence. In the case of Jaffe et al. [63] the explanation of externalizing responses (violence as appropriate conflict resolution, sexism, gender roles in power inequality, etc.) is given from an approach based on modelling and identification with their mother or father. Jouriles et al. [66] explain the higher rate of externalizing responses in girls than boys due to the gender socialization process. They show how the fact that their socialization is “oriented towards others” (care, emotional recognition, etc.), making them more aware of the consequences of violence, and therefore, they adopt more aggressive and oppositional behaviour.

Items 9 and 10 refer to the global analysis of the study. Specifically, the first addresses whether the study helps to increase knowledge of the mental health of girls or boys and diversity in its expression, with a success rate of 7% [56,63,66]. In the last item, research assesses if it helps to highlight changes in gender structure that may affect equality or equity in mental health between boys and girls who have experienced parental gender-based violence. In this case, the success rate is 9% (*n* = 4). Hiltz-Hymes [60] expresses the importance of working on gender roles in interventions with these children, aiming to, without relying on rigid gender roles, develop respect for themselves and others, as well as helping to deconstruct the negative self-image brought about from experiences of violence. On the other hand, Jaffe et al. [63] propose training the various agents who may have contact with these children (teachers, police, etc.) for detection and development of preventive programs, and express the need to continue research to assess the differential impact of group therapy in boys and girls. Suderman et al. [84] consider that, given the results obtained, their intervention is a way of changing attitudes and beliefs about abuse of women, abuse of partners and other forms of violence. Finally, Jouriles et al. [66] highlight how the visits with the aggressor variable can have several consequences in the development of behaviour problems, especially in girls, and the importance of reducing aggression in contacts. Among the different preventive measures, they suggest training of mothers, fathers, social agents (e.g., courts) and limitation of contacts with the aggressor.

### 4.4. Invisibility of Gender-Based Violence

From the total number of studies analysed, only two used the term “gender-based violence” to refer to violence exerted on mothers [77,80]. In most cases (89%), a generic term was used, specifically “intimate partner violence” in 39% of cases and “domestic violence” in 32%, the rest being general terms with few repetitions. These data agree with the results of López-Cepero et al. [15], where the use of general terms was also found to refer to gender violence in the partner; in their case, “domestic violence” was most used. Another aspect to take into account is that although all research included in our analysis includes mothers and their children, only 41% state that violence is received mostly by women or exercised mainly by men. In other words, most studies still make the sex and gender component in violence invisible, either by action (by using general terms) or omission (by omitting information).

Other factors detected showing the invisibility of gender in intimate partner violence are ambiguity when describing the sex of the attacked parent as “parent” and conducting research with mixed groups of fathers and mothers. Such studies comprised around 40% of records excluded in the last screening phase of the study (*n* = 19). It is striking that, of studies excluded by mixed samples of fathers, the percentage of assaulted mothers ranges between 81% and 98%. Nevertheless, exposure to violence is made from general terminology without mentioning gender. Only one item of research stated that the sample is mixed but did not provide data on the number or percentage of fathers and mothers [99]. However, data were provided on the frequency of the perpetrator of intimate partner violence, 65% by the father, 2.5% the mother and 32% both parents (not specifying whether in defence or the frequency of each parent). It is noticeable that in 87% of cases, the mother reported the child’s condition. By not providing data on the number of fathers and mothers, it is possible that this inequality is due to a high number of mothers in the intervention, which would evidence the invisibility of the number of women. In the case of an equal number of mothers and fathers, it would be explained how gender roles put the mother in charge of the care of the children, highlighting one inequality factor.

### 4.5. Programs for Abusive Fathers

Of the total number of studies selected, only one was found where the intervention was with the father and assesses the impact of the intervention on the children’s mental health. This is the quasi-experimental study by Satyanarayana et al. [81], where an intervention was carried out to reduce the father’s alcohol consumption in heteronormative families where gender violence occurred. In their hypotheses, they consider alcohol consumption a gender violence moderator causing distress to the children. Thus, they observe that lower alcohol intake will cause a reduction in gender-based violence and therefore children’s distress. In their results, they found statistically significant differences in the reduction in partner violence between the group receiving the intervention and the group that did not; however, this did not produce any change in the children’s distress. It should be noted that this study did not score on any of the GPIHR items; therefore, interpretation of its results lacks a gender perspective. Likewise, other studies were found where the father received the intervention, but these were discarded because results focused on recurrence of assaults on women, and not on the implications that this intervention might have on children’s mental health.

An explanation of the difference in scientific production regarding evaluation of the consequences of intervention on children to a greater extent in mothers than in fathers may have to do with gender roles. Care is a traditionally feminine area, where the mother is expected to provide for the children. In the same regard, the tendency to not include men in the caregiving role, and only link them to aggression, alcohol consumption and violence, ignores the pedagogical impact of the father in the lives of children. Obviously, interventions must be different for the aggressor and the victim; however, it is often clear that both are parents.

### 4.6. Temporal Evolution of Studies and the Gender Perspective

If we look at the temporal evolution of the studies in the analysis, we find a direct relationship between research methodology factors and publication timing. In other words, a significant relationship has been found regarding the greater timeliness of the publication, the greater frequency of definition of the term of violence used, and the more often that term is defined, the more the gender component in intimate partner violence is exposed. Therefore, it appears there is a certain tendency to operationalise concepts; however, the terms used remain general.

When analysing the possible existence of a relationship between temporality and the gender perspective by decade of publication, we found significant differences both in the total scores of the GPIHR and in the factor “Feminist research”. Generally, these results indicate a temporal evolution of GP in “U”-shaped investigations. In other words, there is a downward trend from publications from before 2000 to the period 2001 to 2010, and subsequently a change with a significant increase from 2011 to 2020 compared to the previous decade. Nonetheless, we must treat these data with caution since, overall, most items answered positively are related to the methodological quality of the study. Therefore, the fact that there are significant differences in the increase at a general level from the decade of 2000 to 2020 does not imply that the investigations generally have GP. In addition, after comparing the “Feminist research” factor, we found significant differences in research prior to 2000 with those carried out between 2001 and 2010, with those prior to 2000 having higher scores.

These results show that the methodological quality of the studies on interventions in minors with experiences of parental gender violence has increased significantly over the years; nevertheless, factors related to the gender perspective have not. This is of vital importance, given that it is a research context with a high gender component, and not having this perspective may bias interpretations of the data.

## 5. Conclusions

Following assessment and analysis of the scientific literature published up to 2020 on interventions with CEXPGBV, we conclude that most studies do not contain a gender perspective. A total of 70% of GPIHR items were answered negatively, and items with the highest success rate related to basic research methodological aspects. Likewise, the lack of a gender perspective in studies and low rates of variables related to gender (term of violence used, specify prevalence of violence against women, etc.) hindered establishing any relationship between the two.

On the other hand, we found a clear difference in compliance of GPIHR between the aspects related to sex and gender. In general, research considered sex at the descriptive level of the sample; however, in most, factors regarding the socialization of gender have been omitted. Likewise, in most studies evaluated, the term gender has been used as a synonym for sex.

In the scientific literature, the operationalization of variables is vital, which is why the indiscriminate use of the terms sex and gender and the use of generic terms to describe gender-based violence hinder inclusion of GP in research in this population. Thus, at present, research faces the challenge of operationalizing social and socialization factors related to gender in childhood, while at the same time continuing to assess components related to the mental health of children. These difficulties might explain the clear trend of the studies towards methodological improvement and paralysis of the evolution of GP in the studies. However, we must bear in mind that methodological quality is not opposed to the gender perspective, both being complementary.

We believe it important to express the methodological limitations of researching the efficacy of interventions in this population. Despite recommendations for the use of RCTs to control variability in the researched condition (focused on internal validity), in CEXPGBV healthcare contexts, this methodology is hardly applicable. Given the high prevalence of intrusive variables (visits with the aggressor, violent episodes during the intervention, changes of address, school, interrupted parental consent, etc.), we found a gap between experimental practice and care practice, since gender-based violence cannot be isolated in the laboratory, nor does it have markers. Faced with this situation, Barkham et al. [100] suggest a complement to the evidence-based practice (characterised by the use of RCTs), practice-based evidence (PBE). In PBE, external validity is prioritised with data from healthcare practice through the routine measurement of responses that generate distress in the person being attended to, in which measurement can be administered pre and post intervention, at repeated intervals or session to session.

Finally, we consider that the GPIHR instrument offers basic aspects for evaluating gender perspective in health research. Among the notable factors, we find: (a) its length of 10 items enables ease of application; (b) applicability in most of the usual research sections; (c) its three dimensions provide clarity on basic components to consider that a study contains GP; (d) ease of adaptation to the population. Likewise, it is not a demanding questionnaire as regards GP in research, but rather offers the basic keys to consider that the research contains GP. Therefore, it is vitally important that future research related to children with experiences of parental gender-based violence take each item of the GPIHR instrument into account both in planning and in elaboration of study.

### Limitations

Despite attempting to achieve the highest representativeness of the published research, we found various limitations. First, heterogeneity of sample. Gender-based violence, being so widespread and varied, means that among included studies we find variations in the recruitment of the sample (women’s shelters, community services, mental health, NGOs). Second, when trying to obtain the largest number of studies by including experimental, quasi-experimental, non-experimental and single-case studies, we found limited control of variability of studies. Third, it has not been analysed whether tests used both in evaluation of distress of the children and of the aggressions in the family context had GP. Fourth, one of the limitations of applying the GPIHR questionnaire is having prior knowledge regarding gender perspective.

## Figures and Tables

**Table 1:** Study characteristics.

Variable	*n*	%
Study design		
Experimental (RCT)	19	42%
Quasi-experimental	13	29%
Non-experimental	11	27%
Single case	1	2%
Violence		
Term of violence with a gender component	5	11%
Definition of the term of violence used	12	27%
Element of sex in violence	18	41%
Assessment of violence	29	64%
Decade		
Up to 2000	5	11%
2001 to 2010	17	39%
From 2011 to 2020	22	50%

**Table 2:** Percentage of compliance with GPIHR items.

Factor	Item	% Compliance
Gender sensitivity	1. In the introduction, have references to existence or non-existence of scientific knowledge with gender perspective been included?	5%
	2. In the introduction, is there any reference to the magnitude of the problem in girls and boys?	30%
	4 Through the objectives/hypotheses formulated, does it seek the association between the health issue studied and any gender determinant?	14%
	7. In the methodology, do the variables used highlight the existing relationship between the health issue studied and any of the gender factor(s): characteristics dependent on the social role, attitudes, beliefs, sex division of work, sexual identity, family role, life	23%
	Total compliance with factor “Sensitivity to gender”	18%
Feminist research	3. Does the introduction take into account the gender category as a health determinant?	7%
	9. Does it aim at helping increase the knowledge of girls and boy’s health and diversity in its expression?	7%
	10. Does it aim at helping point out changes in the gender structure that may affect equality or equity between boys and girls in health?	9%
	Total compliance with the factor “Feminist research”	8%
	5. In methodology, has the sample been stratified by sex?	84%
Sex difference	6. In methodology, has the sample been stratified by age group?	95%
	8. Does the project help bring out the differences or inequalities between boys and girls in the health issue studied?	25%
	Total compliance with factor “Sex difference”	68%

**Table 3:** Probability levels (p) associated with the Kruskal–Wallis test.

	Gender Sensitivity	Feminist Research	Sex Difference	Total Score GPIHR
Study type	0.685	0.711	0.707	0.768
Gender concept in violence	0.456	0.591	0.721	0.402
Definition of the term of violence used	0.616	0.702	0.709	0.627
Element of sex in violence	0.349	0.298	0.244	0.860
Assessment of violence	0.529	0.859	0.035	0.653
Decade of study	0.110	0.045	0.062	0.041

**Table 4:** Spearman’s correlation coefficients.

	Year	Experimental Model	Gender in Terms of Violence	Define Violence	Sex in Intimate Partner Violence	Assessment of Violence	Gender Sensitivity	Gender Sensitivity	Sex Difference	Total Score
Year	1									
Experimental model	0.054	1								
Gender in terms of violence	−0.082	0.129	1							
Define violence	0.417 **	−0.000	−0.058	1						
Sex in intimate partner violence	0.199	0.159	0.139	0.321 *	1					
Assessment of Violence	0.369 *	−0.389 *	−0.027	0.039	−0.236	1				
Gender sensitivity	0.088	0.161	0.114	0.077	0.143	−0.096	1			
Feminist research	−0.097	0.147	0.082	−0.058	0.159	−0.027	0.537 **	1		
Sex difference	0.366 *	−0.076	0.054	0.057	−0.178	0.321 *	0.506 **	0.067	1	
Total score	0.139	0.099	0.128	0.074	0.027	0.069	0.899 **	0.899 **	0.745 *	1

Note. * = The correlation is significant at the 0.05 level (bilateral); ** = The correlation is significant at the 0.01 level (bilateral).

## Data Availability

Publicly available datasets were analyzed in this study. This data can be found here: Web of Science: https://www.webofscience.com; EBSCOhost; https://search.ebscohost.com; ProQuest: https://www.proquest.com; Cochrane Library databases: https://www.cochranelibrary.com.

## Appendix A

**Table A1:** Search elements for systematic review.

Search Element	Keywords Used in Databases
Minors ^a^	child* or “young” or “adolescence” or “adolescent” or teen* or “infant” or “youth” or “minors” or “minor” or son* or daughter* or “young adult”
Psychopathology/symptomatology	“disorder” or emotion* or behavior* or behaviour* or problem* or symptom* “internalizing” or depress* or “anxiety” or “anxious” or “withdrawn” or “somatic” or “social problems” “externalizing” or “agressive” or “defiant” or “attention problems” or “rule-braking” or “disruptive” or “PTSD” or “posttraumatic stress disorder” or “post-traumatic stress disorder” or “trauma” or “transdiagnostic”
Gender Violence ^a^	“intimate partner violence” or “IPV” or “gender violence” or “domestic violence” or abuse* or “violence toward woman” or “marital violence” or victim* or mother* or partner* or spouse* or “wives” or “wife” or parent* or father* or famil*
Psychological Treatment	“treatment” or “psychological treatment” or “intervention” or psycholog*
Efficacy	“evidence” or “strength” or “effectiveness” or “efficacy” or “quality” or “results” or “outcomes”
Studies	“randomized controlled trial” or quasi-experimental stud* or non-experimental stud* case–control stud* or cohort stud* or follow-up stud* or “cross-sectional” or stud* or observational stud*

Note. ^a^ = In Title.

**Table A2:** Items adapted from the questionnaire “Gender perspective in health research” (GPIHR) [8].

Item
1. In the introduction, have references been included to the existence or not of scientific knowledge with a gender perspective? 2. In the introduction, is there any reference to the magnitude of the problem in girls and boys? 3. Does the introduction take into account the gender category as a determinant of health? 4. Through the objectives/hypotheses formulated, do you look for the association between the health problem studied and some gender determinant? 5. In the methodology, has the sample been stratified by sex? 6. In the methodology, has the sample been stratified by age group? 7. In the methodology, do the variables used highlight the relationship between the health problem studied and any of the gender factors: characteristics dependent on social role, attitudes, beliefs, sexual division of labor, sexual identity, family role, cycle vital? 8. Does the project contribute to highlighting differences or inequalities between boys and girls in the health issue investigated? 9. Does it aim to help increase awareness of girls ‘or boys’ health and diversity in its expression? 10. Does it aim to help point out changes in the gender structure that may have an impact on equality or equity, between boys and girls, in health?

Note. The questionnaire “Gender perspective in health research” (GPIHR) [8] comes from an open access article under the CC BY-NC-ND license.

**Table A3:** Selected Studies and GPIHR Scores.

Study	GPIHR Items									
	1	2	3	4	5	6	7	8	9	10
Basu et al. (2009)	0	0	0	0	1	1	0	0	0	0
Cohen et al. (2011)	0	0	0	0	1	1	0	0	0	0
Dodd (2009)	0	0	0	0	0	0	0	0	0	0
Ducharme et al. (2000)	0	0	0	0	1	1	0	0	0	0
Ernst et al. (2007)	0	1	0	0	1	1	1	1	0	0
Galano (2018)	0	1	0	0	1	1	0	1	0	0
Galano et al. (2019)	0	1	0	0	1	1	0	0	0	0
Ghosh et al. (2011)	0	0	0	0	1	1	0	0	0	0
Graham-Berman (2007)	0	1	0	0	1	1	1	1	0	0
Graham-Berman et al. (2011)	0	1	0	1	1	1	1	1	1	0
Graham-Berman et al. (2015)	0	1	0	0	1	1	0	1	0	0
Grip et al. (2011)	0	0	0	0	1	1	0	0	0	0
Grip et al. (2013)	0	0	0	0	1	1	1	1	0	0
Hiltz-Hymes (2011)	1	1	1	1	1	1	0	0	0	1
Howell et al. (2013)	0	1	0	0	1	1	0	1	0	0
Howell et al. (2015)	0	0	0	0	1	1	1	0	0	0
Jaffe et al. (1986)	0	1	1	1	0	1	0	0	1	1
Jouriles et al. (2001)	0	0	0	0	1	1	0	0	0	0
Jouriles et al. (2009)	0	0	0	0	1	1	0	0	0	0
Jouriles et al. (2018)	1	1	1	1	1	1	1	1	1	1
Kearney et al. (2012)	0	0	0	0	0	1	0	0	0	0
Kot et al. (1998)	0	1	0	0	1	1	0	0	0	0
Levitan (2014)	0	0	0	0	1	1	0	0	0	0
Lieberman et al. (2005)	0	0	0	0	1	1	0	0	0	0
Lieberman et al. (2006)	0	0	0	0	1	1	0	0	0	0
Martín-Rodríguez et al. (2002)	0	1	0	0	1	1	0	0	0	0
McDonald et al. (2006)	0	0	0	0	1	1	0	0	0	0
McFarlane et al. (2005)	0	1	0	0	1	1	0	0	0	0
McWhirter (2008)	0	0	0	0	0	1	0	0	0	0
McWhirter (2010)	0	0	0	0	0	1	0	0	0	0
Miller (2012)	0	0	0	0	1	1	0	0	0	0
Muela et al. (2019)	0	1	0	1	1	1	1	1	0	0
Pernebo et al. (2018)	0	0	0	0	1	1	0	1	0	0
Pernebo et al. (2019)	0	0	0	1	1	1	0	1	0	0
Riquelme-Soto et al. (2019)	0	0	0	0	1	1	0	0	0	0
Satyanarayana (2016)	0	0	0	0	0	0	0	0	0	0
Smith et al. (2003)	0	0	0	0	1	1	0	0	0	0
Smith (2016)	0	0	0	0	1	1	0	0	0	0
Suderman et al. (2000)	0	0	0	0	1	1	1	0	0	1
Sullivan et al. (2002)	0	0	0	0	1	1	0	0	0	0
Sullivan et al. (2004)	0	0	0	0	0	1	1	0	0	0
Timmer et al. (2010)	0	0	0	0	1	1	0	0	0	0
Tyndall-Lind et al. (2001)	0	0	0	0	1	1	0	0	0	0
Wagar et al. (1995)	0	0	0	0	1	1	1	0	0	0
Waldman-Levi et al. (2015)	0	0	0	0	1	1	0	0	0	0
